# Biodistribution and Toxicity Studies of PRINT Hydrogel Nanoparticles in Mosquito Larvae and Cells

**DOI:** 10.1371/journal.pntd.0003735

**Published:** 2015-05-21

**Authors:** Yashdeep Phanse, Brendan M. Dunphy, Jillian L. Perry, Paul M. Airs, Cynthia C. H. Paquette, Jonathan O. Carlson, Jing Xu, J. Christopher Luft, Joseph M. DeSimone, Barry J. Beaty, Lyric C. Bartholomay

**Affiliations:** 1 Department of Entomology, Iowa State University, Ames, Iowa, United States of America; 2 Lineberger Comprehensive Cancer Center, University of North Carolina at Chapel Hill, North Carolina, United States of America; 3 Department of Microbiology, Immunology, and Pathology, Colorado State University, Fort Collins, Colorado, United States of America; 4 Eshelman School of Pharmacy, University of North Carolina at Chapel Hill, Chapel Hill, North Carolina, United States of America; 5 Department of Chemistry, University of North Carolina at Chapel Hill, Chapel Hill, North Carolina, United States of America; 6 Institute for Nanomedicine and Institute for Advanced Materials, University of North Carolina at Chapel Hill, Chapel Hill, North Carolina, United States of America; 7 Department of Chemical and Biomolecular Engineering, North Carolina State University, Raleigh, North Carolina, United States of America; 8 Sloan-Kettering Institute for Cancer Research, Memorial Sloan-Kettering Cancer Center, New York, New York, United States of America; National Institute of Allergy and Infectious Diseases, UNITED STATES

## Abstract

Mosquito-borne diseases continue to remain major threats to human and animal health and impediments to socioeconomic development. Increasing mosquito resistance to chemical insecticides is a great public health concern, and new strategies/technologies are necessary to develop the next-generation of vector control tools. We propose to develop a novel method for mosquito control that employs nanoparticles (NPs) as a platform for delivery of mosquitocidal dsRNA molecules to silence mosquito genes and cause vector lethality. Identifying optimal NP chemistry and morphology is imperative for efficient mosquitocide delivery. Toward this end, fluorescently labeled polyethylene glycol NPs of specific sizes, shapes (80 nm x 320 nm, 80 nm x 5000 nm, 200 nm x 200 nm, and 1000 nm x 1000 nm) and charges (negative and positive) were fabricated by Particle Replication in Non-Wetting Templates (PRINT) technology. Biodistribution, persistence, and toxicity of PRINT NPs were evaluated *in vitro* in mosquito cell culture and *in vivo* in *Anopheles gambiae* larvae following parenteral and oral challenge. Following parenteral challenge, the biodistribution of the positively and negatively charged NPs of each size and shape was similar; intense fluorescence was observed in thoracic and abdominal regions of the larval body. Positively charged NPs were more associated with the gastric caeca in the gastrointestinal tract. Negatively charged NPs persisted through metamorphosis and were observed in head, body and ovaries of adults. Following oral challenge, NPs were detected in the larval mid- and hindgut. Positively charged NPs were more efficiently internalized *in vitro* than negatively charged NPs. Positively charged NPs trafficked to the cytosol, but negatively charged NPs co-localized with lysosomes. Following *in vitro* and *in vivo* challenge, none of the NPs tested induced any cytotoxic effects.

## Author Summary

There is an urgent need for new interventions and novel insecticides to control mosquito vectors of human disease agents. Nanoparticle-based strategies have been explored extensively as means to deliver a molecule of interest for medical applications (e.g., antigens, drugs, nucleic acids, etc.) to particular tissues and cells in mammalian systems. Our particular application of interest is to use nanoparticles as a platform to deliver nucleic-acid based mosquitocidal molecules. As a first step to developing nanoparticle delivery for molecular vector control, we investigated the physicochemical properties of PRINT hydrogel nanoparticles with respect to their biodistribution, persistence and safety in *Anopheles gambiae* mosquito larvae and in cell culture. This technology could be applied to studies of pathobiology and physiology in any vector species, with the benefit that the particles can be tailored to move to and target specific delivery of a cargo to a particular organ system and even sub-cellular location. Therefore, this technology and specific information about where the particles go and with what affinity sets the stage of describing a platform that has tremendous potential use in vector control and disease transmission intervention.

## Introduction

Vector-borne diseases continue to be major causes of morbidity and mortality worldwide [1]. Malaria remains a major burden on humankind; more than 3 billion people are at risk for infection and more than 200 million people per year are infected, resulting in more than 600,000 reported deaths annually [2]. There has been a dramatic reduction in malaria mortality in the last decade, especially in sub-Saharan Africa and principally attributable to the use of long lasting insecticide treated bed nets and indoor residual spraying (IRS) [2]. Unfortunately these dramatic gains in public health are threatened by the emergence of insecticide resistance, particularly pyrethroid resistance, in mosquito vector populations [3]. Insecticide resistance has emerged to most classes of chemical insecticides, and no new insecticides have been licensed or approved for large-scale application in decades [4]. There is a compelling need to develop new insecticidal interventions and approaches for control of mosquito vectors and the pathogens they transmit.

RNA interference (RNAi)-based technologies are a promising means to induce lethality in pest insect populations by down-regulating physiologically essential genes [5,6]. For example, oral administration of dsRNA in the western corn rootworm, *Diabrotica virgifera* LeConte, induces larval stunting and mortality [7]. However, major challenges need to be overcome to make this technology broadly applicable for molecular insecticide delivery. First, there is wide variability in the successful application of RNAi for gene silencing in insects, in part as a function of whether exposure to an RNAi trigger induces a localized (i.e., tissue-specific) or systemic effect [6,8,9]. Second, dsRNA must persist in the harsh conditions that exist both in the environment and *in vivo* where dsRNA is subject to degradation by enzymes in the body of the insect [10,11]. An efficient delivery vehicle that can enhance dsRNA environmental stability, delivery (orally or contact), internalization, and RNAi efficiency of would be of enormous value for control of insect pests including vectors. All of these efficiencies could result into dose-sparing of dsRNA, an important component of the cost of goods for successful development of intervention strategies. Chitosan NPs have been used to deliver dsRNAs and siRNAs to silence host genes in *An*. *gambiae* and *Ae*. *aegypti* mosquitoes [12,13], but there is much to be learned regarding physicochemical characteristics of NP for optimized stability and delivery to control pest insects including vector species.

NPs have been explored extensively to deliver associated/encapsulated biomolecules such as siRNA, DNA and antigens for a broad spectrum of medical applications [14–16]. Initiatives, such as encapsulation of insecticides, are already underway to prolong their lifespan during indoor residual spraying (IRS) [17]. For therapeutics, NPs fabricated by Particle Replication in Nonwetting Templates (PRINT) technology show superior promise as delivery agents due to their controlled size, shape, and composition [18–23]. Furthermore, as a proof-of-principle for this application, PRINT particles have proven efficacy in delivery of nucleic acid [24,25] and small molecule cargoes [26–29]. Therefore, we reasoned that PRINT NP technology could be combined with RNAi to develop a new generation of effective, safe, and target-specific insecticides for control of both juvenile and adult *An*. *gambiae*.

To determine optimal NP physicochemical characteristics for delivery of mosquitocidal dsRNA molecules for control of juvenile *An*. *gambiae* mosquitoes, larvae and mosquito cells were exposed to PRINT polyethylene glycol-based hydrogel NPs of defined shape, size, and surface charge. Particle surface charge was varied by utilizing either hydroxyl or amine functionalized monomers to yield negative or positive particles respectively. The biodistribution of the NPs in orally challenged *Anopheles gambiae* larvae and internalization efficiencies and pathways in target mosquito cells were determined as a first step toward NP delivery of mosquitocides to juvenile *An*. *gambiae*.

## Materials and Methods

### NP Manufacture and Characterization

#### Materials

Poly(ethylene glycol) diacrylate (Mn 700) (PEG700DA), 2-aminoethyl methacrylate hydrochloride (AEM), and diphenyl (2,4,6-trimethylbenzoyl)-phosphine oxide (TPO) were purchased from Sigma-Aldrich. Thermo Scientific Dylight 488, PTFE syringe filters (13 mm membrane, 0.22 μm pore size), sterile water, and methanol were obtained from Fisher Scientific. Conventional filters (2 μm) were purchased from Agilent, polyvinyl alcohol (Mw 2000) (PVOH) was purchased from Acros Organics, and Luvitec (MW 64 kDa) was purchased from BASF. PRINT molds (80 nm x 320 nm, 80 nm x 5000 nm, and 200 nm x 200 nm) were obtained from Liquidia Technologies. Tetraethylene glycol monoacrylate (HP4A) was synthesized in-house as previously described [30].

#### Methods

PRINT particle fabrication technique has been described previously in detail [19,31]. The pre-particle solution was prepared by dissolving 3.5 wt% of the various reactive monomers in methanol. The reactive monomers included: a cure-site monomer (an oligomeric PEG with a nominal molar mass of 700 g/mol terminally functionalized on both end groups with an acryloxy functionality); a hydrophilic monomer used to make up the majority of the particle composition (HP4A); an amine containing monomer (AEM) which served to provide a positive charge; and a polymerizable fluorescent tag (Dylight 488). In all cases a photoinitiator, TPO, was also added. Two different pre-particle solutions were used throughout the following studies. For negatively charged particles the pre-particle solution was comprised of 88 wt% HP4A, 10 wt% PEG700DA, 1 wt% Dylight maleimide 488, and 1 wt% TPO. For positively charged particles, the pre-particle solution was comprised of 68 wt% HP4A, 20 wt% AEM, 10 wt% PEG700DA, 1 wt% Dylight maleimide 488, and 1 wt% TPO. Using a # 3 Mayer rod (R.D. Specialties), a thin film of the pre-particle solution was drawn onto a roll of freshly corona treated PET, using a custom-made roll-to-roll lab line (Liquidia Technologies) running at 12 ft/min. The solvent was evaporated from this delivery sheet by exposing the film to a hot air dam derived from heat guns. The delivery sheet was laminated (80 PSI, 12 ft/min) to the patterned side of the mold, followed by delamination at the nip. Particles were cured by passing the filled mold through a UV-LED (Phoseon, 395 nm, 3 SCFM N2, 12 ft/min). Either a PVOH (for positively charged particles) or a Luvitech (for negatively charged particles) harvesting sheet was hot laminated to the filled mold (140 °C, 80 PSI, 12 ft/min). Upon cooling to room temperature, particles were removed from the mold by splitting the harvesting sheet from the mold. Particles were then harvested by dissolving the harvesting film in a bead of water (1 mL of water per 5 ft of harvesting sheet). The particle suspension was passed through a 2 μm filter (Agilent) to remove any large particulates. To remove the excess harvesting material, particles were centrifuged (Eppendorf Centrifuge 5417R) at ca. 21,000 g for 15 min, the supernatant was removed and the particles were re-suspended in sterile water. This purification process was repeated 4 times.

### Nanoparticle Characterization

Stock particle concentrations were determined by thermogravimetric analysis (TGA) using a TA Instruments Q5000 TGA. TGA analysis was conducted by pipetting 20 μL of the stock NP solution into a tared aluminum sample pan. Samples suspended in water were heated at 30 °C/min to 130 °C, followed by a 10 minute isotherm at 130 °C, then cooled at 30 °C/min to 30 °C, followed by a 2 minute isotherm at 30 °C. TGA was also performed on a 20 μL aliquot of supernatant from a centrifuged sample of the stock solution to account for the mass of any stabilizer remaining in each sample. The concentration of stabilizer was subtracted from the concentration of stock particle solution to determine the actual particle concentration.

### Mosquito Life Cycle and Larval Rearing

*Anopheles gambiae* G3 strain mosquitoes were reared at 27°C and 70% humidity. Eggs were collected from ovipositional dishes 3 days after bloodfeeding and transferred to a pan of deionized water for hatching the following day. Larvae were distributed to a density of 300 per pan and fed ground TetraMin Tropical Flakes Fish Food (Tetra, Blacksburg, VA) at all instars in the following amounts: 1st instars—5 drops plus a pinch sprinkled on water surface; 2nd instars—10 drops daily; 3rd instars—15 drops daily; 4th instars—20 drops daily until peak pupation was reached.

### Cellular Uptake

To evaluate the internalization of NPs, C6/36 cells (*Aedes albopictus* larval cells) were plated in Liebovitz’s L-15 media (fetal bovine serum (FBS, 10%), penicillin-streptomycin (1%) and L-glutamine (1%)) at density of 0.5 x10^6^ cells/mL in a 24-well plate with coverslips and incubated overnight at 28°C. The following day, cells were incubated with DyLight 488 labeled NPs (15 μg/mL) for 24 h. Cells were then washed with phosphate buffered saline (PBS, pH 7.4) to remove non-adherent or loosely adherent NPs and fixed in 4% paraformaldehyde (methanol free). Cells were permeabilized with 0.1% Triton X-100 in PBS for 3 min and washed with PBS. Actin staining was performed by incubating cells with Alexa Fluor 546 Phalloidin (Life Technologies, NY) for 20 min in PBS at room temperature [32]. Coverslips containing stained cells were washed and mounted on glass slides using ProLong with DAPI (Life Technologies, NY). Confocal microscopy was performed using an inverted Olympus Fluoview 1000 laser scanning microscope. Final images were prepared using Image J v1.47m software (NIH, Bethesda, MD).

### Lysosomal Localization

4a-3b cells (*Anopheles gambiae* larval cells) were plated in Schneider’s media with fetal bovine serum (FBS, 10%), at density of 0.25 x10^6^ cells/mL in a 24-well plate with coverslips and incubated overnight at 28°C. The following day, cells were incubated with DyLight 488 labeled NPs (15 μg/mL) for 12 h. Cells were then washed with phosphate buffered saline (PBS, pH 7.4) to remove non-adherent or loosely adherent NPs and incubated with 100 nM LysoTracker Red DND-99 (Molecular Probes, Invitrogen) containing media for 3 h. Following the incubation, cells were fixed with 4% paraformaldehyde, and mounted on glass slides using ProLong Gold with DAPI (Life Technologies, NY). Confocal microscopy was performed using an inverted Olympus Fluoview 1000 laser scanning microscope. Images were prepared using Image J v1.47m software (NIH, Bethesda, MD).

### In Vitro Cytotoxicity Assay

C6/36 cells were seeded in a 96-well microtiter plate at a density of 1.6x 10^5^ cells/well in Leibovitz-15 media (10% fetal bovine serum, 1% Penicillin/Streptomycin and 1% L-glutamine) for 16 h at 28°C. Hydrogel NPs were added to cells at 250 μg/mL and incubated for 2 h and 48 h at 28°C. Following the incubation, 15 μL of dye solution [CellTiter 96 Non-Radioactive cell proliferation assay MTT (3-(4,5-dimethylthiazol-2-yl)-2,diphenyltetrazoliumbromide) (Promega)] was added and cells were further incubated for 3.5 h at 28°C. After the incubation, 100 μL of solubilization solution was added and cells were incubated for 1 h at room temperature. The optical density (OD) was measured at 570 nm with a background subtraction at 650 nm. Cells not incubated with NPs were used as controls. Cell viability is presented as a percentage of OD_(570–650)_Experimental/ OD_(570–650)_Control.

### Biodistribution and Mortality Studies in Larvae

Larvae were transferred individually to filter paper as means to immobilize them for injections. Individuals were viewed with a stereo-microscope and, using a Nanoject Microneedle ID Injection (Drummond Scientific Company, Broomall, PA) and micromanipulator to position the capillary needle, larvae were injected with 45 nL of nuclease-free water or NP (5 mg/mL) solution through the dorsal arthrodial membrane (mid-sagittal axis) joining the head and thorax. Injection needles were changed for each treatment group.

Larvae were dissected into head, thorax, abdomen, fore- and mid- and hindgut regions. Images were captured at 0, 24, 48, 72 h post- injection. Images of adults injected during their larval stage were also collected post-pupation. Epifluorescence microscopy was performed using a Nikon microscope equipped with red, green and blue filter sets and a cooled CCD camera. Final images were prepared using Image J v1.47m software (NIH). For mortality studies, fourth instars were injected with NPs as mentioned above. Survivorship was recorded every day through adulthood (6 post-injection).

### Per Os Administration of NPs

A mixture of NPs, ground Tetramin (Tetra, Blacksburg, VA) fish food and agarose was prepared by mixing 25 μL (5 mg/mL) NPs with 25 μL Tetramin slurry that then was mixed with 100 μL molten agarose (1%). Blocks of agarose were introduced into cartons containing larvae. 3–5 larvae were randomly selected at each time point for dissection and subsequent microscopic analysis.

### In Vivo Imaging

Fourth instar *An*. *gambiae* were injected with NPs. Larvae were placed in a drop of PBS on a glass slide under light microscope. Heads were removed using a forceps. The thorax was then held in place with one forceps as the gut was removed from the body by gently pulling and removing the last abdominal segment. Dissected tissues were placed on an imaging tray. The tissues were imaged with an *In vivo* Multispectral FX Pro imaging system (Carestream, Rochester, NY, USA) using 480 nm excitation and 535 nm emission wavelengths. Fluorescence measurements were performed at the same exposure setting to compare all data sets to each other. A white light image was also captured to define the tissue boundaries and regions of interest. Image Analysis NIH Image Jv1.47m was utilized to quantify the mean fluorescent intensities (MFI) values. A region of interest (ROI) was drawn around each tissue utilizing the white light image. The same ROI was then applied to the fluorescent image and MF quantified utilizing the Analyzex→Measure function. To account for the fact that the different particle groups have different inherent fluorescence, a correction factor was applied to the raw MFI. The correction factor was calculated as the ratio of fluorescent intensity of the particle group to the intensity value of the brightest particle group (80 nm x 320 nm negative). The resulting number was then divided by the raw MFI of each individual to obtain a corrected MFI value (cMFI). Data for *in vivo* imaging were log transformed.

### Statistical Analysis

Statistical analysis was performed using JMP software (SAS Institute, Cary, NC). Comparisons between treatments were made by Tukey’s HSD (honest significant difference). Differences were considered significant for p < 0.05.

## Results

### Internalization of Hydrogel NPs In Vitro

**Fig 1:**
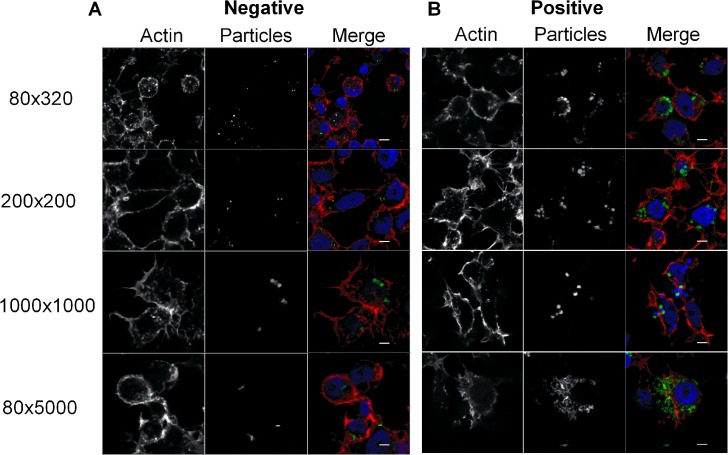
Efficient internalization of PRINT particles by C6/36 cells. Cell monolayers were incubated with Dylight 488 labeled (A) negative or (B) positive particles (green) for 24 h and stained for actin (Alexa phalloidin 546, red). DAPI (blue) was used to stain the nucleus. Representative images were captured by LSCM. Scale bars = 5 μm.

In order to be effective, the delivery vehicle of choice should be efficiently internalized by target cells. Size, shape and charge play a crucial role in the internalization of NPs [33]. To test whether the PRINT hydrogel NPs are internalized by insect cells and if the physicochemical properties play a role, *Aedes albopictus* C6/36 cells were incubated with fluorescently labeled NPs and evaluated for internalization by fluorescence microscopy. NPs of all sizes and charge were taken up efficiently (Fig 1). Positively charged NPs were more efficiently internalized than negatively charged NPs (Fig 1). The differences could be dramatic, for example, when cells were challenged with 80 nm x 5000 nm positively charged NPs, intense fluorescence was detected in the cells; in contrast there was only low-level fluorescence detected in cells challenged with the negatively charged NPs (Fig 1). Similar results were observed when NPs were incubated with *An*. *gambiae* cell lines 4a3a and 4a-3B (S2 and S3 Figs).

### Cellular Trafficking

**Fig 2:**
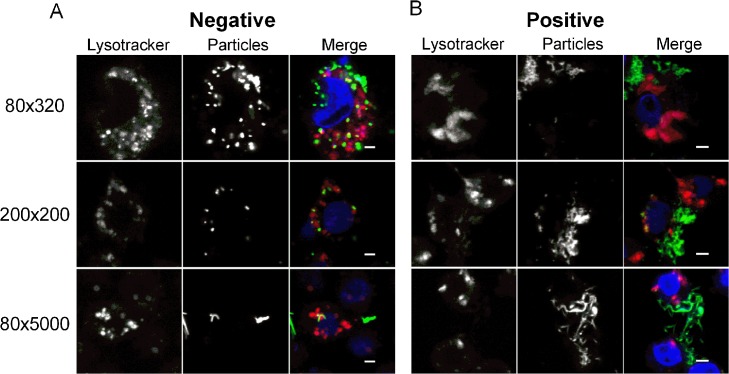
Positive PRINT particles escape trafficking to lysosomal compartments. Cell monolayers were incubated with Dylight 488 labeled (A) negative or (B) positive particles (green) for 24 h and stained for acidic organelles i.e., lysosomes (Lysotracker Red DND, red). DAPI (blue) was used to stain the nucleus. Representative images were captured by LSCM. Scale bars = 2 μm.

To activate the RNAi response efficiently, we reasoned that the NPs should deliver the dsRNA cargo to cytosol where it can be incorporated into the RISC complex. The trafficking of NPs to lysosomal compartments may result in degradation of dsRNA. To identify the trafficking patterns of PRINT NPs, 4a-3B cells were exposed to the respective NPs, fixed, stained, and visualized using confocal microscopy (Fig 2). The cellular biodistribution of the positively and negatively charged NPs were strikingly different. The majority of 80 nm x 320 nm and 200 nm x 200 nm negatively charged NPs co-localized with acidic organelles, e.g., lysosomes (Fig 2A). In contrast, positively charge NPs were detected principally in the cytosol, indicating that they either avoided or escaped lysosomal compartments of cells (Fig 2B). Interestingly, the 80 nm x 5000 nm negative NPs and were not co-localized with the lysosomes, similar to positively charged NPs (Fig 2A).

### Biodistribution

**Fig 3:**
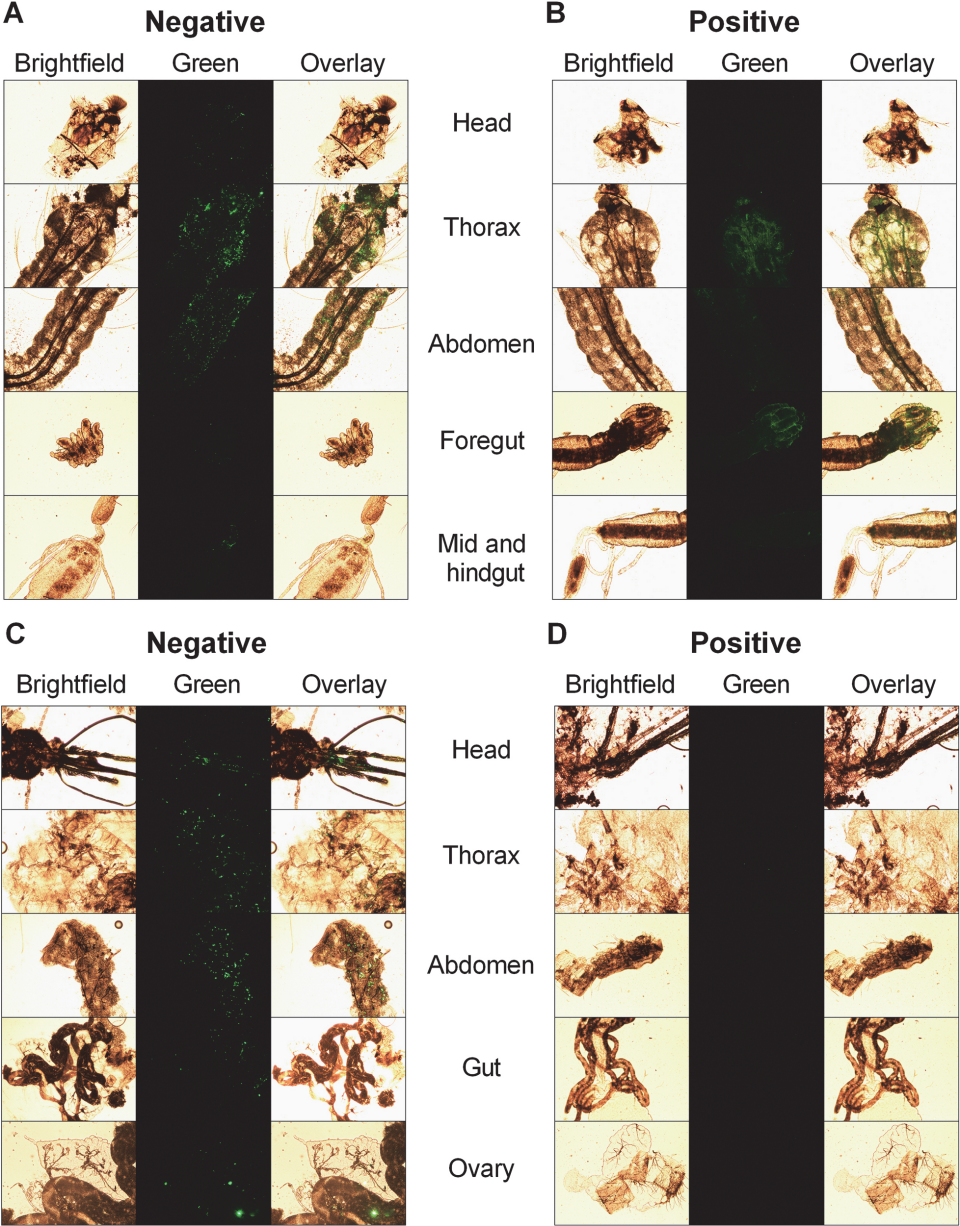
Biodistribution of 80 nm x 5000 nm DyLight 488 labeled hydrogel particles in *An*. *gambiae* larvae and adults. Fourth instar larvae were injected with 45 nL (235 ng) of 80 nm x 5000 nm (A) negative and (B) positive charged particles. Larvae were dissected into head, thorax, abdomen, foregut and mid and hindgut regions. Images were captured at 24 h post-injection. Adults emerging from larvae that were injected with 80 nm x 5000 nm (C) negative and (D) positive particles were imaged day 1 post eclosion. Data shown are representative of results obtained from three independent experiments. Images were captured at 100X magnification.

To determine the biodistribution of NPs *in vivo*, *An*. *gambiae* larvae were parenterally challenged with the respective NPs, dissected into head, thorax, and abdomen regions, and further to fore-, mid- and hindgut sections of the gastrointestinal tract at day 0, 1, 2 and 3 post-injection (p.i.), and examined by fluorescence microscopy to determine the biodistribution of the respective NPs. A striking difference was observed in the biodistribution of negatively and positively charged NPs; the former showed an intense punctate staining while the latter exhibited diffuse fluorescence (Fig 3A and 3B). This biodistribution of positively and negatively charged particles was also seen in parenterally challenged adult mosquitoes (see Figs 10 and 13 in companion paper) [34]. In the head, NPs of all sizes and both charges were observed through day 3 p.i., although the fluorescence signal with negatively charged NPs was greater than that with positively charged NPs (Figs 3 and S1). The most intense fluorescence for both positively and negatively charged NPs was observed in thorax and abdominal segments of the larval body. Here too, negatively charged NPs showed punctate fluorescence while positively charged NPs presented as more diffuse (Fig 3A and 3B). nterestingly, positively charged NPs of all sizes were more often associated with gastric caeca in the foregut than the negatively charged NPs (Fig 3B).

Adults emerging from larvae injected with NPs were also evaluated for the persistence of NPs during metamorphosis. Negatively charged NPs persisted through metamorphosis and were present in the head, thorax, abdomen and gut of adult female mosquitoes at day 1 post-emergence (Fig 3C). Ovaries were also imaged to determine the possibility of vertical transmission of NPs. Negatively charged NPs of all sizes were observed in follicles and tracheae of the ovaries (Fig 3C). 80 nm x 5000 nm negatively charged NPs were also present in the maxillary palps and proboscis and other tissues in the head (Fig 3C). Negatively charged particles were much more likely to be associated with these tissues than positively charged particles in adult mosquitoes (see Figs 7 and 11 in the companion paper) [34].

### Oral Challenge

**Fig 4:**
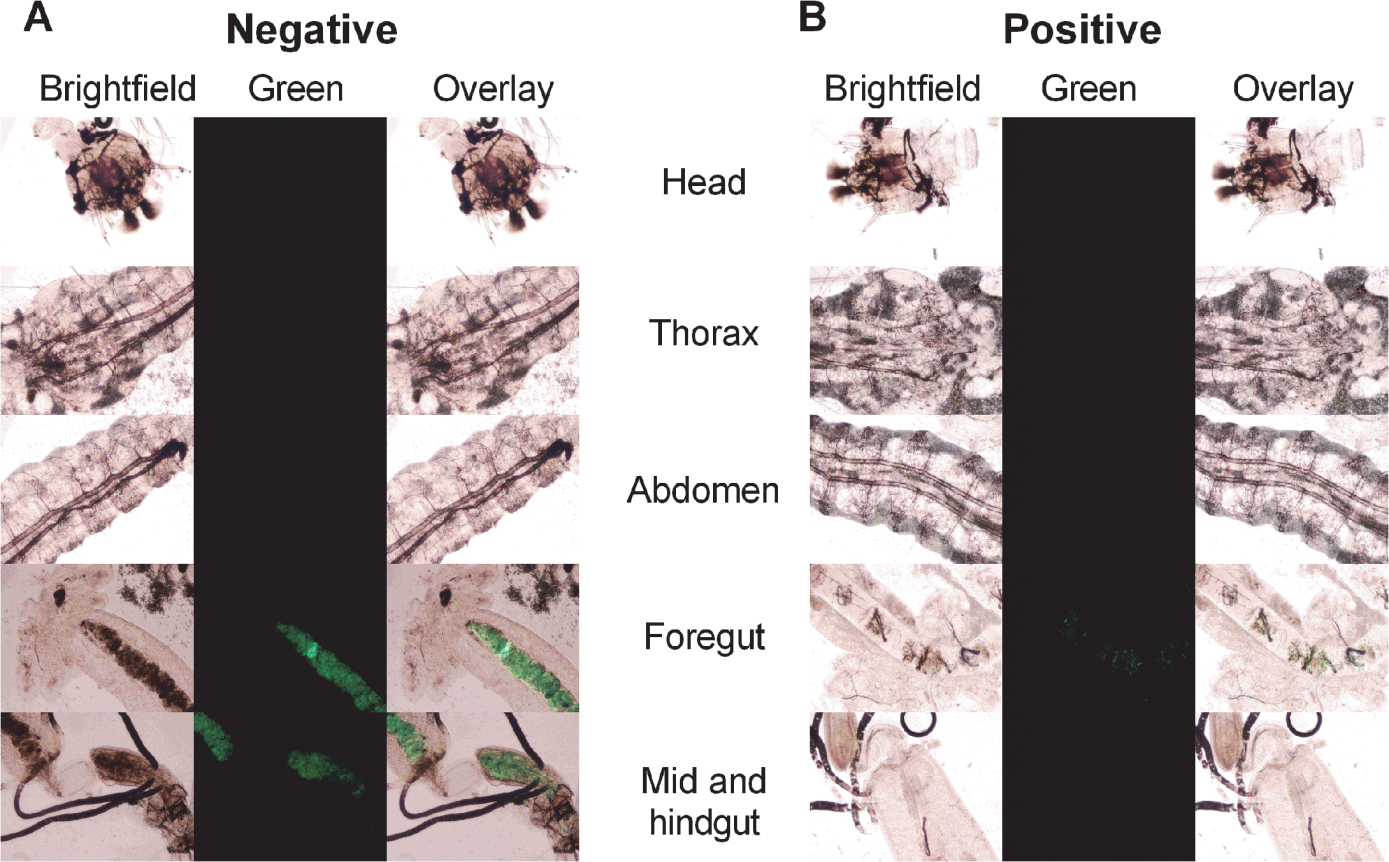
Biodistribution of 80 nm x 5000 nm DyLight 488 labeled hydrogel particles in *An*. *gambiae* larvae post *per os* administration. Fourth instar larvae were exposed to 80 nm x 5000 nm particles mixed in tetramin food and 1% agarose. (A) negative and (B) positive charged particles. Larvae were dissected into head, thorax, abdomen, foregut and mid and hindgut regions. Images were captured at 120 h post-exposure. Data shown are representative of results obtained from three independent experiments. Images were captured at 100X magnification.

Field-applicable control of larvae necessitates gut or transcuticular delivery of NPs loaded with dsRNA. In a proof-of-concept experiment, *An*. *gambiae* were exposed to PRINT 80 nm x 5000 nm NPs mixed in larval food and agarose. The larvae readily ingested the formulation containing NPs (Fig 4). NPs were observed in fore-, mid- and hindgut. However, no fluorescence was observed outside of the lumen of the digestive tract (Fig 4).

### In Vivo Imaging

**Fig 5:**
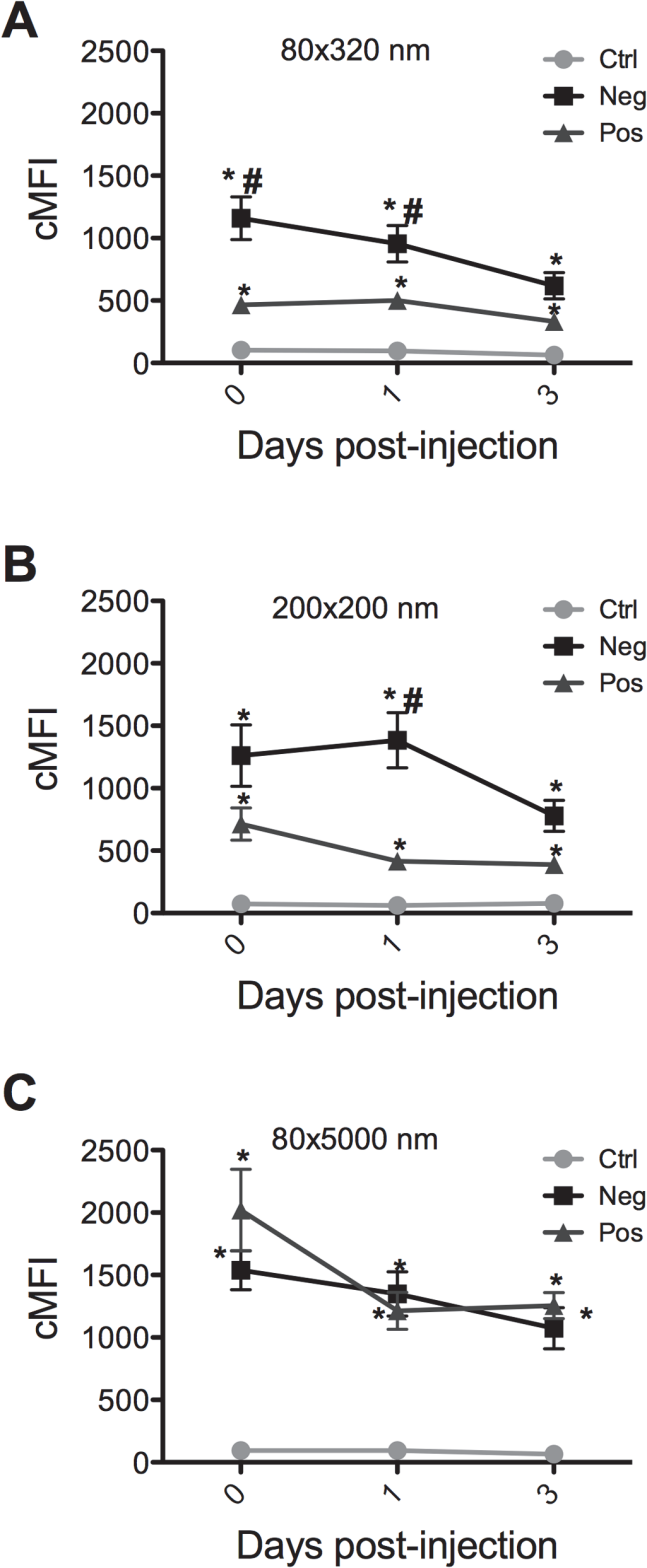
*In vivo* fluorescence quantification of particles. Fourth instar larvae were injected with 45 nL (235 ng) (A) 80 nm x 320 nm (B) 200 nm x 200 nm and (C) 80 nm x 5000 nm particles. At indicated time points, larvae were dissected and fluorescence from thorax and abdomen quantified. Data are expressed as the mean ± SEM of three independent experiments. n = 10 larvae/group/time point. Statistical difference (p<0.05) from controls and positives is indicated by * and #, respectively.

For efficient, systemic RNAi, dsRNA should reach and persist in the hemolymph where it will be distributed throughout the body by virtue of circulation through the open circulatory system [35]. Studies have shown that dsRNA is enzymatically degraded in hemolymph of some insects e.g., *Manduca sexta* [11]. Thus, to increase hemolymph delivery, the NP vehicle should protect the dsRNA when trafficking in the larval body. In this study, *in vivo* imaging was employed to test the persistence of hydrogel NPs in larval thoraces and abdomens. All of the particle groups tested persisted through 3 days p.i. (Fig 5). Interestingly, the 80 nm x 320 nm and 200 nm x 200 nm negatively charged NPs were more abundantly detected at each time point than positively charged NPs. This difference was not as great with the 80 nm x 5000 nm NPs. This may be attributable to more efficient internalization of the positively charged NPs in mosquito cells *in vivo* as was shown *in vitro* (Fig 1).

### Cell Viability Analysis

**Fig 6:**
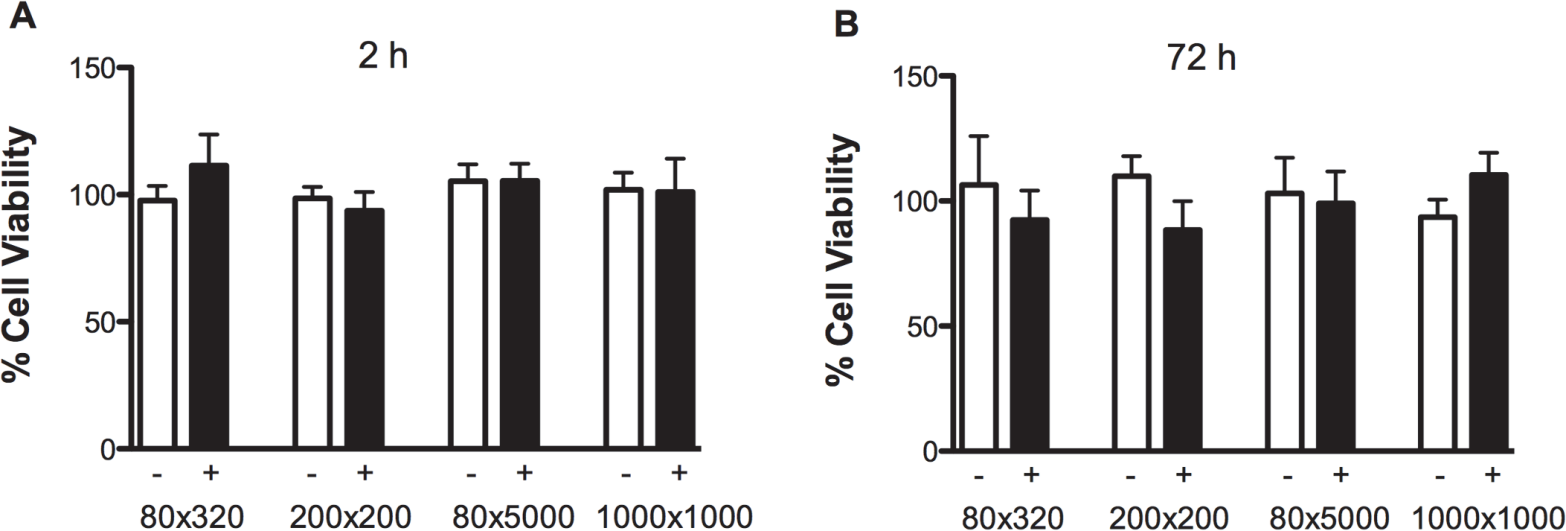
*In vitro* safety profiling of PRINT particles on C6/36 insect cells. Cell monolayers were exposed to negative (open bar) or positive (closed bars) particles at a concentration of 250 μg/mL for (A) 2 h and (B) 72 h. Results reported are averages of three independent experiments performed in triplicate. Untreated cells were used as controls for 100% cell viability. No statistical difference (p<0.05) was observed for any of the particle treatment groups compared to untreated controls.

To determine if the NPs themselves have inherent cytotoxicity in mosquito cells, C6/36 cells were incubated with NPs and cell viability was tested at 2 and 72 h post-incubation by MTT assay. None of the particle groups exhibited any cytotoxic effects on C6/36 cells at any of the indicated time points (Fig 6A and 6B). Interestingly, 80 nm x 5000 nm NPs, which were internalized most efficiently, also demonstrated an excellent cell viability profile.

### Survivorship

**Fig 7:**
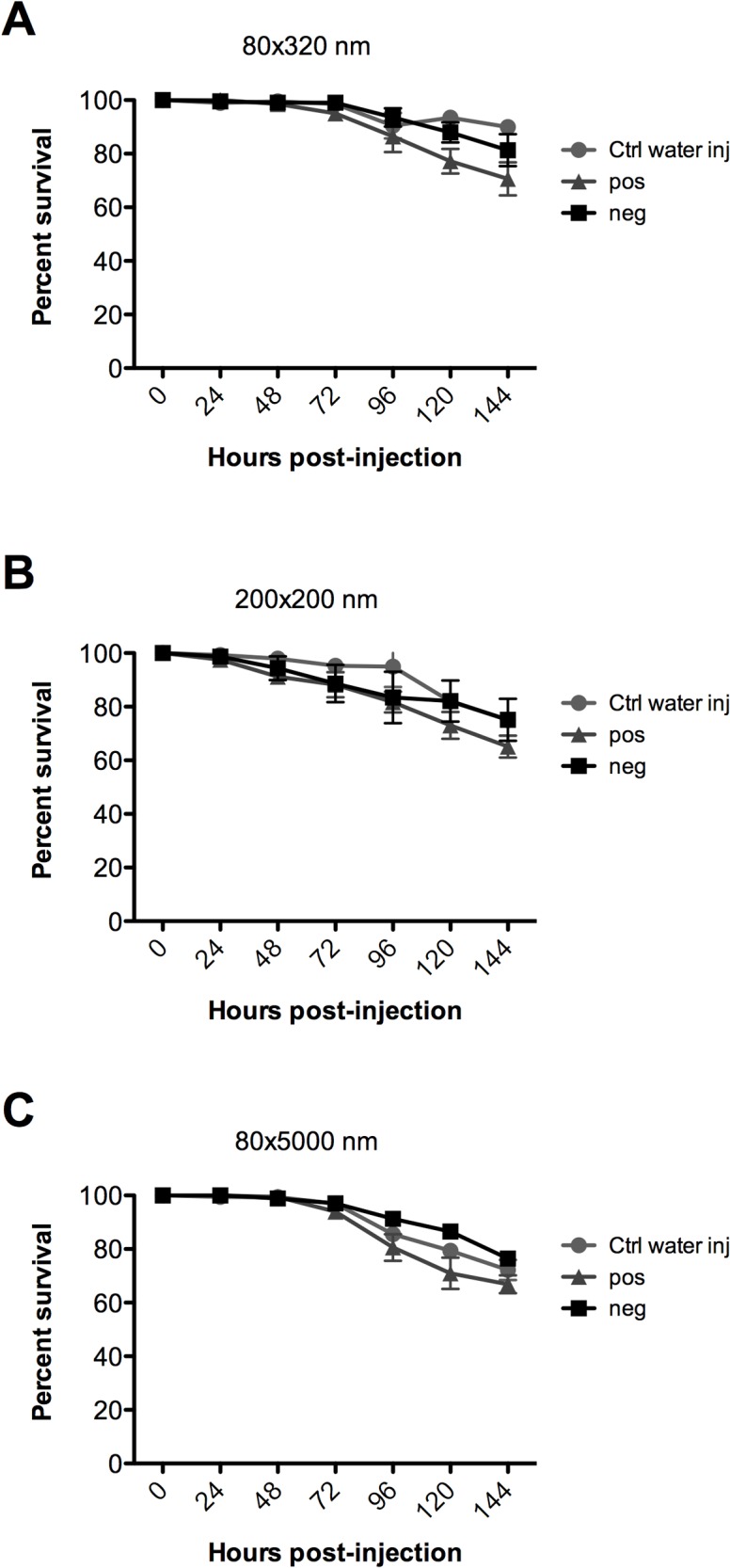
Survivorship of *An*. *gambiae* larvae after injection with particles. Fourth instar larvae were injected with 45 nL (235 ng) (A) 80 nm x 320 nm (B) 200 nm x 200 nm and (C) 80 nm x 5000 nm particles. Larval survival was monitored through 144 h post-injection. Data are expressed as the mean ± SEM of five independent experiments. No statistical difference between groups was observed at p<0.05.

Complementing the *in vitro* cell viability studies, *in vivo* studies were conducted to determine if the NPs caused untoward effects in *An*. *gambiae* larvae. Fourth instar larvae were intrathoracically injected with NPs and their survival monitored for 6 days p.i., Larvae injected with water were used as controls. No significant mortality was observed by any of the NP groups studied compared to controls (Fig 7). The low levels of mortality observed in larvae injected with water are likely caused by the stress of injection. Together, these data show that PRINT NPs themselves do not induce any untoward effects in *An*. *gambiae* larvae.

## Discussion

Novel vector control strategies are critically needed to combat emerging and re-emerging vector-borne diseases. Alternative approaches such as dsRNA induced RNAi mediated lethality have great potential for vector control. Critical steps in RNAi-based approaches are 1) environmental stability and delivery of the RNAi trigger, and 2) uptake of the dsRNA by target cells. Our data show that NPs of all sizes and charges are efficiently internalized by mosquito cells both in culture and *in vivo* (Fig 1). Previous work has shown that positively charged NPs are internalized more efficiently than negatively charged NPs in mammalian cells because of their electrostatic interactions with negatively charged plasma membrane [36–38]. Similar results were obtained with mosquito cells, in which positively charged PRINT NPs are internalized better than the negatively charged NPs (Fig 1). In addition to charge, size and shape of NPs also can play an important role in uptake. Indeed, positive NPs of 80 nm x 320 nm, 200 nm x 200 nm and 80 nm x 5000 nm were internalized more readily than 1000 nm x 1000 nm positive NPs (Fig 1).

Once the delivery vehicle is internalized, it is crucial that the payload (e.g., dsRNA) is delivered to the cytosol where the RNAi machinery is located and not to the lysosomes where it could get degraded. Lysosomal trafficking studies revealed that size and charge of the NPs is important in the trafficking events post-internalization by cells. A majority of negatively charged 80 nm x 320 nm and 200 nm x 200 nm NPs trafficked to lysosomes whereas positively charged NPs of all sizes were not co-localized with lysosomes (Fig 2). Positively charged NPs also showed a diffuse fluorescence pattern inside cells that suggests presence in cytosol. These data suggest that positively charged NPs might be better candidate(s) than negatively charged NPs to deliver dsRNA triggers. To the best of our knowledge, this is the first study showing the effect of particle charge, shape and size on internalization and trafficking in insect cells.

A critical step during *in vivo* dsRNA-mediated gene silencing is association of the RNAi trigger with specific tissues in the host body. Biodistribution studies of PRINT NPs were performed to study the effect of size and charge on spatiotemporal distribution. Negatively charged NPs of all sizes exhibited intense punctate fluorescence (Fig 3A) whereas positively charged NPs had a diffuse fluorescence pattern (Fig 3B). This could be due to more efficient internalization of positively charged NPs leading to loss of fluorescence *in vivo*. Because both negatively and positively charged NPs were present in head, thorax and abdomen, NPs are likely distributed throughout the body via hemolymph (Fig 3). Interestingly, positively charged NPs exhibit a strong localization affinity toward the gastric caeca (Fig 3B). A possible explanation could be the faster attachment and/or internalization of positively charged NPs on the above-mentioned tissues. Thus, positively charged NPs might function better to deliver dsRNA to silence genes expressed specifically in gut tissue. Malpighian tubules did not contain detectable NPs indicating that these NPs may not be the suitable delivery candidates to target this organ system. In contrast, NPs were detected abundantly in malpighian tubules of parenterally but not orally challenged adult mosquitoes [34]. Very interestingly, NPs (principally negatively charged) persisted through metamorphosis to the adult stage in mosquitoes that matured from larvae that were injected with NPs (Fig 3C and 3D). Negatively charged NPs were detected in to the thorax, abdomen, ovaries, proboscis and other head tissues (Fig 3C and 3D). Presence of NPs in the latter tissues suggests exciting potential for the vertical transmission to subsequent generation as well as via contact spread. The physiologic mechanism(s) that facilitate NP persistence through metamorphosis remain to be determined.

Oral feeding is the simplest and most field applicable way of environmentally delivering dsRNA to insects; however, the efficiency of inducing RNAi orally is poor [39]. The proof-of-concept *per os* delivery experiments indicated that larva could feed on PRINT NPs mixed in food slurry and agarose. Twenty four hours after exposure, NPs were observed in fore, mid-and hindgut regions of the larval digestive tract (Fig 4). It is possible that NPs tested in this study did not traffic into the body because the agarose matrix in which the NP were delivered did not release particles in the gut, or because the larval peritrophic matrix (PMII) presents an impermeable barrier.

NP fluorescence was quantified in thorax and abdomen of parenterally challenged larvae. The fluorescence signal of negatively charged 80 nm x 320 nm and 200 nm x 200 nm NPs was greater than that with positively charged NPs, most notably at day 0 and day 1 p.i. (Fig 5A and 5B). The fluorescence signal of the negatively charged NPs declined with time from day 0 to day 3 p.i. (Fig 5). The fluorescent signal with 80 nm x 320 nm positively charged NPs was lower but more stable through day 3 p.i. (Fig 5A). The 80 nm x 5000 nm NPs differed from other NPs; the signal from positively charged NPs was greater than negatively charged NPs at day 0 and day 1 p.i. (Fig 5B and 5C). It is likely that the differences observed in fluorescence intensity over time are a function of cellular uptake and trafficking of the particles as revealed in three separate mosquito cell lines. It is noteworthy that the positively and negatively charged particles exhibited exactly the same phenotype following parenteral challenge of adult mosquitoes (see Fig 4 in the companion paper) [34].

In order for an insecticide delivery system to be viable, it is key that it not be inherently cytotoxic. Previous studies showed that these particular particles demonstrate high levels of cellular internalization in mammalian cells, with minimal cytotoxicity [40]. PRINT particles are non-toxic in vitro in multiple human cancer cell lines [24,25,29,40,41]. We have also evaluated PRINT particles in vivo in mouse models and shown that they are non-toxic and do not induce an inflammatory response [42]. In keeping with these data, mosquito cell viability assays showed that PRINT NPs did not induce any undesired toxic effects in mosquito cells after 2 and 72 hour incubation (Fig 6). To further validate the *in vitro* results, 4^th^ instar larvae were injected with PRINT NPs and their survival monitored through day 6 p.i. (Fig 7). None of the particle groups studied caused difference in survivorship from the water injected controls, and thus had no adverse effect on larval development, pupation, and emergence to the adult stage.

In conclusion, we report the detailed biodistribution and viability evaluations of PRINT NPs in mosquito cells and in *An*. *gambiae* larva as the first step in rational design of molecular mosquitocides. The excellent, low cell and larval toxicity profiles, efficient internalization, and widespread biodistribution make these NPs attractive candidates for dsRNA delivery in mosquitoes. The presence of NPs in head and ovaries may be indicators of contact uptake and vertical transmission capabilities, respectively. These attributes could be exploited to control adult as well as larval mosquitoes. Nanotechnology mediated delivery of mosquitocides offers a new paradigm for designing next-generation vector control tools.

## Supporting Information

S1 Fig Biodistribution of 200 nm x 200 nm and 80 nm x 320 nm DyLight 488 labeled hydrogel particles in *An*. *gambiae* larvae and adults. Fourth instar larvae were injected with 45 nL (235 ng) of 200 nm x 200 nm (A) negative and (B) positive or 80 nm x 320 nm (C) negative and (D) positive charged particles. Larvae were dissected into head, thorax, abdomen, foregut and mid and hindgut regions. Images were captured at 24 h post-injection. Data shown are representative of results obtained from three independent experiments. Images were captured at 100X magnification.
(TIF)

S2 Fig Efficient internalization of PRINT particles by 4a3Acells. Cell monolayers were incubated with Dylight 488 labeled (A) negative or (B) positive particles (green) for 24 h and stained for actin (Alexa phalloidin 546, red). DAPI (blue) was used to stain the nucleus. Representative images were captured by LSCM. Scale bars = 5 μm.
(TIF)

S3 Fig Efficient internalization of PRINT particles by 4a-3B cells. Cell monolayers were incubated with Dylight 488 labeled (A) negative or (B) positive particles (green) for 24 h and stained for actin (Alexa phalloidin 546, red). DAPI (blue) was used to stain the nucleus. Representative images were captured by LSCM. Scale bars = 5 μm.
(TIF)

## References

[1] Gubler DJ (2009) Vector-borne diseases. Rev Sci Tech 28: 583–588. 20128467 10.20506/rst.28.2.1904

[2] WHO (2013) World Malaria Report.

[3] Hemingway J (2014) The role of vector control in stopping the transmission of malaria: threats and opportunities. Philos Trans R Soc Lond B Biol Sci 369: 20130431 10.1098/rstb.2013.0431 24821917 PMC4024224

[4] Hemingway J, Beaty BJ, Rowland M, Scott TW, Sharp BL (2006) The Innovative Vector Control Consortium: improved control of mosquito-borne diseases. Trends Parasitol 22: 308–312. 16713358 10.1016/j.pt.2006.05.003

[5] Price DR, Gatehouse JA (2008) RNAi-mediated crop protection against insects. Trends Biotechnol 26: 393–400. 10.1016/j.tibtech.2008.04.004 18501983

[6] Huvenne H, Smagghe G (2010) Mechanisms of dsRNA uptake in insects and potential of RNAi for pest control: a review. J Insect Physiol 56: 227–235. 10.1016/j.jinsphys.2009.10.004 19837076

[7] Baum JA, Bogaert T, Clinton W, Heck GR, Feldmann P, Ilagan O, et al (2007) Control of coleopteran insect pests through RNA interference. Nat Biotechnol 25: 1322–1326. 17982443 10.1038/nbt1359

[8] Scott JG, Michel K, Bartholomay LC, Siegfried BD, Hunter WB, Smagghe G, et al (2013) Towards the elements of successful insect RNAi. J Insect Physiol 59: 1212–1221. 10.1016/j.jinsphys.2013.08.014 24041495 PMC3870143

[9] Whangbo JS, Hunter CP (2008) Environmental RNA interference. Trends Genet 24: 297–305. 10.1016/j.tig.2008.03.007 18450316

[10] Guo P, Haque F, Hallahan B, Reif R, Li H (2012) Uniqueness, advantages, challenges, solutions, and perspectives in therapeutics applying RNA nanotechnology. Nucleic Acid Ther 22: 226–245. 10.1089/nat.2012.0350 22913595 PMC3426230

[11] Garbutt JS, Belles X, Richards EH, Reynolds SE (2013) Persistence of double-stranded RNA in insect hemolymph as a potential determiner of RNA interference success: evidence from *Manduca sexta* and *Blattella germanica* . J Insect Physiol 59: 171–178. 10.1016/j.jinsphys.2012.05.013 22664137

[12] Zhang X, Zhang J, Zhu KY (2010) Chitosan/double-stranded RNA nanoparticle-mediated RNA interference to silence chitin synthase genes through larval feeding in the African malaria mosquito (*Anopheles gambiae*). Insect Mol Biol 19: 683–693. 10.1111/j.1365-2583.2010.01029.x 20629775

[13] Mysore K, Flannery EM, Tomchaney M, Severson DW, Duman-Scheel M (2013) Disruption of *Aedes aegypti* olfactory system development through chitosan/siRNA nanoparticle targeting of semaphorin-1a. PLoS Negl Trop Dis 7: e2215 10.1371/journal.pntd.0002215 23696908 PMC3656119

[14] Yu YH, Kim E, Park DE, Shim G, Lee S, Kim YB, et al (2012) Cationic solid lipid nanoparticles for co-delivery of paclitaxel and siRNA. Eur J Pharm Biopharm 80: 268–273. 10.1016/j.ejpb.2011.11.002 22108492

[15] Perez SE, Gandola Y, Carlucci AM, Gonzalez L, Turyn D, Bregni C (2012) Formulation strategies, characterization, and *in Vitro* evaluation of lecithin-based nanoparticles for siRNA delivery. J Drug Deliv 2012: 986265 10.1155/2012/986265 22570790 PMC3335242

[16] Phanse Y, Carrillo-Conde BR, Ramer-Tait AE, Broderick S, Kong CS, Rajan K, et al (2014) A systems approach to designing next generation vaccines: combining alpha-galactose modified antigens with nanoparticle platforms. Sci Rep 4: 3775 10.1038/srep03775 24441019 PMC3895907

[17] Oxborough RM, Kitau J, Jones R, Feston E, Matowo J, Mosha FW, et al (2014) Long-lasting control of *Anopheles arabiensis* by a single spray application of micro-encapsulated pirimiphos-methyl (Actellic(R) 300 CS). Malar J 13: 37 10.1186/1475-2875-13-37 24476070 PMC3914366

[18] Wang J, Byrne JD, Napier ME, DeSimone JM (2011) More effective nanomedicines through particle design. Small 7: 1919–1931. 10.1002/smll.201100442 21695781 PMC3136586

[19] Perry JL, Herlihy KP, Napier ME, Desimone JM (2011) PRINT: a novel platform toward shape and size specific nanoparticle theranostics. Acc Chem Res 44: 990–998. 10.1021/ar2000315 21809808 PMC4157651

[20] Perry JL, Reuter KG, Kai MP, Herlihy KP, Jones SW, Luft JC, et al (2012) PEGylated PRINT nanoparticles: the impact of PEG density on protein binding, macrophage association, biodistribution, and pharmacokinetics. Nano Letters 12: 5304–5310. 10.1021/nl302638g 22920324 PMC4157665

[21] Galloway AL, Murphy A, DeSimone JM, Di J, Herrmann JP, Hunter ME, et al (2013) Development of a nanoparticle-based influenza vaccine using the PRINT technology. Nanomedicine 9: 523–531. 10.1016/j.nano.2012.11.001 23178283

[22] Xu J, Wong DH, Byrne JD, Chen K, Bowerman C, DeSimone JM (2013) Future of the particle replication in nonwetting templates (PRINT) technology. Angew Chem Int Ed Engl 52: 6580–6589. 10.1002/anie.201209145 23670869 PMC4157646

[23] Xu J, Luft JC, Yi X, Tian S, Owens G, Wang J, et al (2013) RNA replicon delivery via lipid-complexed PRINT protein particles. Mol Pharm 10: 3366–3374. 10.1021/mp400190z 23924216 PMC3948333

[24] Dunn SS, Tian SM, Blake S, Wang J, Galloway AL, Murphy A, et al (2012) Reductively responsive siRNA-conjugated hydrogel nanoparticles for gene silencing. Journal of the American Chemical Society 134: 7423–7430. 10.1021/ja300174v 22475061 PMC3357068

[25] Hasan W, Chu K, Gullapalli A, Dunn SS, Enlow EM, Luft JC, et al (2012) Delivery of multiple siRNAs using lipid-coated PLGA nanoparticles for treatment of prostate cancer. Nano Letters 12: 287–292. 10.1021/nl2035354 22165988 PMC3358784

[26] Chu KS, Finniss MC, Schorzman AN, Kuijer JL, Luft JC, Bowerman CJ, et al. (2014) Particle replication in nonwetting templates nanoparticles with tumor selective alkyl silyl ether docetaxel prodrug reduces toxicity. Nano Letters. 10.1021/nl4046558 PMC4157645 24552251

[27] Chu KS, Hasan W, Rawal S, Walsh MD, Enlow EM, Luft JC, et al (2013) Plasma, tumor and tissue pharmacokinetics of Docetaxel delivered via nanoparticles of different sizes and shapes in mice bearing SKOV-3 human ovarian carcinoma xenograft. Nanomedicine-Nanotechnology Biology and Medicine 9: 686–693. 10.1016/j.nano.2012.11.008 23219874 PMC3706026

[28] Chu KS, Schorzman AN, Finniss MC, Bowerman CJ, Peng L, Luft JC, et al (2013) Nanoparticle drug loading as a design parameter to improve docetaxel pharmacokinetics and efficacy. Biomaterials 34: 8424–8429. 10.1016/j.biomaterials.2013.07.038 23899444 PMC3807740

[29] Parrott MC, Finniss M, Luft JC, Pandya A, Gullapalli A, Napier ME, et al (2012) Incorporation and controlled release of silyl ether prodrugs from PRINT nanoparticles. Journal of the American Chemical Society 134: 7978–7982. 10.1021/ja301710z 22545784 PMC3362319

[30] Guzmán MTI J., Riande E., Compañ V., Andrio A. (1997) Synthesis and polymerization of acrylic monomers with hydrophilic long side groups. Oxygen transport through water swollen membranes prepared from these polymers. Polymer 38: 5227–5232.

[31] Merkel TJ, Herlihy KP, Nunes J, Orgel RM, Rolland JP, DeSimone JM (2010) Scalable, shape-specific, top-down fabrication methods for the synthesis of engineered colloidal particles. Langmuir 26: 13086–13096. 10.1021/la903890h 20000620 PMC2891593

[32] Phanse Y, Ramer-Tait AE, Friend SL, Carrillo-Conde B, Lueth P, Oster CJ, et al. (2012) Analyzing cellular internalization of nanoparticles and bacteria by multi-spectral imaging flow cytometry. J Vis Exp: e3884. 10.3791/3884 PMC3471309 22710268

[33] Verma A, Stellacci F (2010) Effect of surface properties on nanoparticle-cell interactions. Small 6: 12–21. 10.1002/smll.200901158 19844908

[34] Paquette CCH, Phanse Y, Perry J, Sachez-Vargas I, Airs PM, Dunphy BM, et al. (accepted 2015) Biodistribution and trafficking of hydrogel nanoparticles in adult *Anopheles gambiae* mosquitoes. PLoS Neglected Tropical Diseases. 10.1371/journal.pntd.0003745 PMC4440717 25996505

[35] Glenn JD, King JG, Hillyer JF (2010) Structural mechanics of the mosquito heart and its function in bidirectional hemolymph transport. J Exp Biol 213: 541–550. 10.1242/jeb.035014 20118304

[36] Phanse Y, Carrillo-Conde BR, Ramer-Tait AE, Roychoudhury R, Pohl NL, Narasimhan B, et al (2013) Functionalization of polyanhydride microparticles with di-mannose influences uptake by and intracellular fate within dendritic cells. Acta Biomater 9: 8902–8909. 10.1016/j.actbio.2013.06.024 23796408

[37] Foged C, Brodin B, Frokjaer S, Sundblad A (2005) Particle size and surface charge affect particle uptake by human dendritic cells in an *in vitro* model. Int J Pharm 298: 315–322. 15961266 10.1016/j.ijpharm.2005.03.035

[38] Wischke C, Borchert HH, Zimmermann J, Siebenbrodt I, Lorenzen DR (2006) Stable cationic microparticles for enhanced model antigen delivery to dendritic cells. J Control Release 114: 359–368. 16889866 10.1016/j.jconrel.2006.06.020

[39] Yu N, Christiaens O, Liu J, Niu J, Cappelle K, Caccia S, et al (2013) Delivery of dsRNA for RNAi in insects: an overview and future directions. Insect Sci 20: 4–14. 10.1111/j.1744-7917.2012.01534.x 23955821

[40] Gratton SE, Ropp PA, Pohlhaus PD, Luft JC, Madden VJ, Napier ME, et al (2008) The effect of particle design on cellular internalization pathways. Proc Natl Acad Sci U S A 105: 11613–11618. 10.1073/pnas.0801763105 18697944 PMC2575324

[41] Wang J, Tian S, Petros RA, Napier ME, DeSimone JM (2010) The Complex role of multivalency in nanoparticles targeting the transferrin receptor for cancer therapies. Journal of the American Chemical Society 132: 11306–11313. 10.1021/ja1043177 20698697 PMC2923393

[42] Roberts RA, Shen T, Allen IC, Hasan W, DeSimone JM, Ting JPY (2013) Analysis of the murine immune response to pulmonary delivery of precisely fabricated nano- and microscale particles. PloS One 8. 10.1371/journal.pone.0062115 PMC3625166 23593509

