# Supplementary figures and images for: Biodistribution and Toxicity Studies of PRINT Hydrogel Nanoparticles in Mosquito Larvae and Cells
Source: PLoS Negl Trop Dis. 2015 May 21;9(5):e0003735. doi: 10.1371/journal.pntd.0003735 (PMC4440723; doi:10.1371/journal.pntd.0003735)

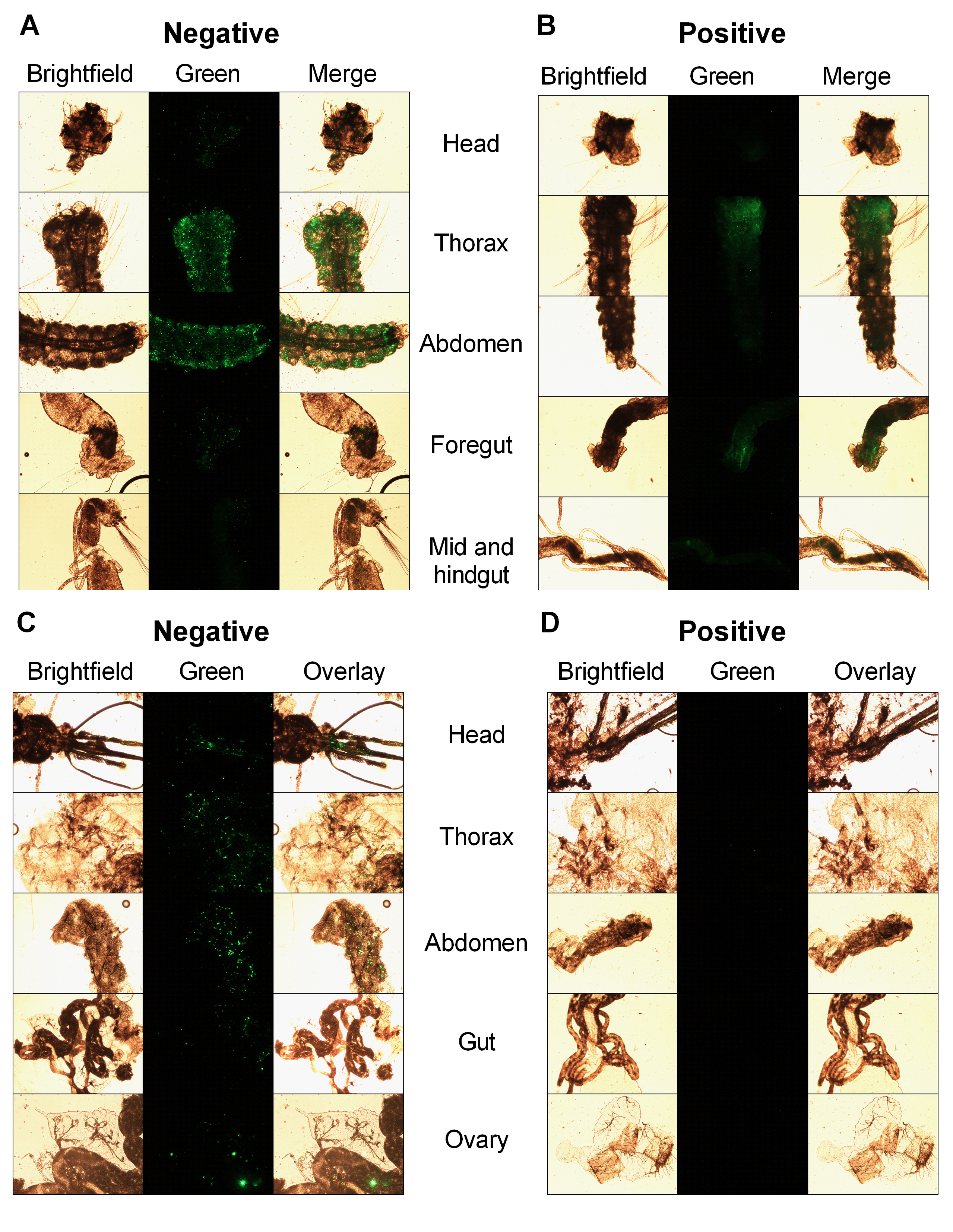

Supplement: S1 Fig — Fourth instar larvae were injected with 45 nL (235 ng) of 200 nm x 200 nm (A) negative and (B) positive or 80 nm x 320 nm (C) negative and (D) positive charged particles. Larvae were dissected into head, thorax, abdomen, foregut and mid and hindgut regions. Images were captured at 24 h post-injection. Data shown are representative of results obtained from three independent experiments. Images were captured at 100X magnification. (TIF) [file pntd.0003735.s001.tif]
